# Antibacterial activity of a modified unfilled resin containing a novel polymerizable quaternary ammonium salt MAE-HB

**DOI:** 10.1038/srep33858

**Published:** 2016-09-23

**Authors:** Li Huang, Fan Yu, Xiang Sun, Yan Dong, Ping-ting Lin, Hao-han Yu, Yu-hong Xiao, Zhi-guo Chai, Xiao-dong Xing, Ji-hua Chen

**Affiliations:** 1 State Key Laboratory of Military Stomatology & National Clinical Research Centre for Oral Diseases & Shaanxi International Joint Research Center for Oral Diseases, Department of General Dentistry and Emergency, School of Stomatology, Fourth Military Medical University, Xi’an, Shaanxi, 710032, P. R. China; 2 State Key Laboratory of Military Stomatology & National Clinical Research Centre for Oral Diseases & Shaanxi key Laboratory of Stomatology, Department of Prosthodontics, School of Stomatology, Fourth Military Medical University, Xi’an, 710032, P. R. China; 3 State Key Laboratory of Military Stomatology & National Clinical Research Centre for Oral Diseases & Shaanxi Engineering Research Center for Dental Materials and Advanced Manufacture, Department of VIP Dental Care, School of Stomatology, Fourth Military Medical University, Xi’an, 710032, P. R. China; 4 Department of Stomatology, Kunming General Hospital of PLA, Kunming, 650032, P. R. China; 5 School of Chemical Engineering, Nanjing University of Science and Technology, Nanjing, 210094, P. R. China

## Abstract

Resins with strong and long-lasting antibacterial properties are critical for the prevention of secondary dental caries. In this study, we evaluated the antibacterial effect and the underlying mechanism of action of an unfilled resin incorporating 2-methacryloxylethyl hexadecyl methyl ammonium bromide (MAE-HB) against *Streptococcus mutans* UA159 (*S. mutans* UA159). MAE-HB was added into unfilled resin at 10 mass%, and unfilled resin without MAE-HB served as the control. Bacterial growth was inhibited on 10%-MAE-HB unfilled resin compared with the control at 1 d, 7 d, 30 d, or 180 d (P < 0.05). The growth inhibitory effect was independent of the incubation time (P > 0.05). No significant differences in the antibacterial activities of eluents from control versus 10%-MAE-HB unfilled resins were observed at any time point (P > 0.05). The number of bacteria attached to 10%-MAE-HB unfilled resin was considerably lower than that to control. Fe-SEM and CLSM showed that 10%-MAE-HB unfilled resin disturbed the integrity of bacterial cells. Expression of the bacterial glucosyltransferases, *gtfB* and *gtfC*, was lower on 10%-MAE-HB unfilled resin compared to that on control (P < 0.05). These data indicate that incorporation of MAE-HB confers unfilled resin with strong and long-lasting antibacterial effects against *S. mutans.*

Dental caries is one of the most prevalent infectious diseases in the world. Resins have been widely used for restoring decayed teeth mainly for their adhesive properties, better aesthetics, and reduced preparation size^1^,^2^,^3^. However, resin restorations are associated with higher failure rates owing to secondary caries^2^,^4^,^5^. The main shortcoming of resin-based materials is the potential for microleakage due to polymerization shrinkage, occlusal forces, and aging, thus enabling for bacterial invasion^6^. In addition, resins accumulate more dental plaque than other materials, which may increase the possibility of bacterial microleakage, leading to restoration failures and pulpal damage^7^,^8^. Therefore, resins with strong and long-lasting antibacterial properties are highly desirable.

Several approaches for endowing dental materials with antibacterial properties have been explored. Examples include modification of resins by addition of soluble antimicrobials such as fluoride, Ag, and chlorhexidine^9^,^10^. The addition of fluoride to resins has been applied widely owing to its beneficial effects in reducing demineralization, enhancing remineralization, and inhibiting microbial metabolism and plaque formation^11^. However, the release of fluoride may be not sufficient for a maximal antibacterial effect^12^. In the case of agent-releasing antibacterial resins, tight control of the release kinetics of the antimicrobials remains a challenge^13^, and the antibacterial activity of modified resins decreases with time^14^,^15^,^16^. Besides, the mechanical or physical properties of the parent resin may be compromised by the constant release of antibacterial agents. This is especially due to the porous surface that is formed during the release process, which may lead to poor wear resistance and increase the potential for staining and bacterial biofilm accumulation^14^. To overcome this issue, research groups have subsequently focused on the development of polymerizable antibacterial agents that are non-volatile.

12-Methacryloyloxy-dodecylpyridiniumbromide (MDPB) and methacryloxyethyl cetyl dimethyl ammonium chloride (DMAE-CB), which were developed by Imazato *et al*.^17^ and by our research group^18^,^19^,^20^, can be chemically immobilized within resins. However, the amount of MDPB or DMAE-CB monomers incorporated into resin materials is limited^19^,^21^. This is because there is only one double bond in their molecular structures, and thus a limited amount of MDPB or DMAE-CB monomers can bind to the resin matrix during polymerization^22^.

To solve this problem, our group synthesized a novel polymerizable quaternary ammonium salt (QAS) monomer, MAE-HB, which has two double bonds^22^. MAE-HB exhibited strong bactericidal activities against *Streptococcus mutans, Actinomyces viscosus, Lactobacillus acidophilus, Staphylococcus aureus, Streptococcus sanguinis, Porphyromonas gingivalis, Prevotella melaninogenica*, and *Enterococcus faecalis*^22^. However, whether resins containing MAE-HB exhibit improved antibacterial activity compared to existing materials remains unclear. In this study, we added MAE-HB to an experimental light-curable unfilled dental resin, and investigated the antibacterial effects and the underlying mechanism of action of this composite.

## Methods

### Preparation of unfilled resins

**Figure 1:**
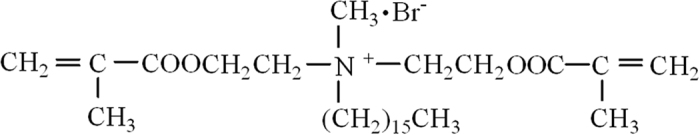
Structure of the QAS monomer, MAE-HB.

The structure of MAE-HB is presented in Fig. 1. The experimental light-curable unfilled resin consisted of 2,2-bis[4-(2-hydroxy-3-methacryloxypropoxy)phenyl] propane (Bis-GMA, Esstech, PA) and triethylene glycol dimethacrylate (TEGDMA, Esstech) with a mass ratio of 75:25. The photosensitizer camphorquinone (Esstech) was added at 0.5 wt%, and dimethylaminoethylmethacrylate (Sigma-Aldrich, St. Louis, MO) was added at 1 wt%. MAE-HB was added as an immobilized bactericide at 10 mass% (hereafter 10%-MAE-HB unfilled resin), and resin without MAE-HB (0%-MAE-HB unfilled resin) served as the control.

Unfilled resins were dropped into stainless steel moulds (10 mm in diameter and 3 mm in thickness). The top and bottom surfaces were covered with a polyester matrix and cured for 60 s with a light activation unit delivering 450 mW/cm2 (Dentsply QHL71, Milford, DE). Then, the unfilled resins were sterilized with ethylene oxide gas, followed by degassing for 48 h.

### Bacterial strain and culture conditions

*S. mutans* UA159 was cultured at 37 °C in brain heart infusion (BHI) broth (Difco, Detroit, MI) in an anaerobic atmosphere (90% N_2_, 5% CO_2_, and 5% H_2_). Then, the overnight culture was adjusted to 1 × 10^5^ colony forming units (CFU)/ml for subsequent experiments.

### Antibacterial activity of immobilized MAE-HB

The film contact method was used to evaluate the antibacterial activity of immobilized MAE-HB^19^. Unfilled resins were immersed in 2 ml distilled water at 37 °C for 1 d, 7 d, 30 d, or 180 d. The distilled water was changed daily. One hundred microliters of the bacterial suspension was dropped onto the specimen surface, which was then covered with a celluloid film and incubated at 37 °C for 24 h in an anaerobic atmosphere. Bacteria were collected by vortexing the specimen in 9.9 ml BHI for 2 min. The bacterial suspension was diluted 10-fold, and 100 μl of bacterial suspension was inoculated onto a BHI agar plate to quantify the number of CFU recovered. Five specimens were tested for each group.

### Antibacterial activity of released MAE-HB

All specimens of 0%-MAE-DB and 10%-MAE-HB unfilled resin were immersed in 2 ml BHI at 37 °C for 1 d, 7 d, 30 d, or 180 d. The BHI was changed daily. The BHI at different immersion times was collected as the eluent and transferred to a 12-well plate. One hundred microliters of bacterial suspension was inoculated into the eluent and incubated at 37 °C for 24 h in an anaerobic atmosphere. Bacterial suspensions from each specimen were diluted 10-fold, and 100 μl was inoculated onto a BHI agar plate to quantify the number of CFU recovered. Five specimens were tested for each group.

### Fe-SEM observation

All specimens of 0%-MAE-DB and 10%-MAE-HB unfilled resin were placed in a 24-well plate with 20 μl of bacterial suspension applied on the top. Two millilitres of BHI supplemented with 1% sucrose was added into each well after incubation at 37 °C for 1 h. After a further 4-h incubation, the specimens with biofilm were gently rinsed with distilled water and fixed in 2.5% glutaraldehyde in 0.1 mol/l cacodylate buffer at pH 7.2 for 4 h at room temperature. Specimens were then dehydrated in an ascending ethanol series with a critical-point drier. After being coated with gold using an ion sputter (JFC-1100E, JEOL, Japan), the central portion of the specimens was observed with Fe-SEM (S-4800, Hitachi, Tokyo, Japan).

### CLSM analysis of bacterial growth

Specimens coated with *S. mutans* were prepared as described above and were analysed by CLSM^23^. After a 24-h incubation, the biofilm-coated disks were washed three times with sterile saline to remove loose bacteria, and the remaining bacteria were stained using the Live/Dead BacLight Bacterial Viability Kit (Cat. No. L7012, Molecular Probes, Eugene, OR, USA) with 15-min incubation in the dark at room temperature to allow stain development prior to image scanning. With this kit, live bacteria are stained by Syto 9 and produce green fluorescence, and bacteria with compromised membranes will be stained by propidium iodide and produce red fluorescence. The samples were rinsed gently with distilled water and observed by CLSM (FluoView FV1000, Olympus, Tokyo, Japan). Excitation with a 488-nm laser revealed the green fluorescence emission of live bacteria, and excitation with a 543-nm laser revealed the red fluorescence emission of bacteria with damaged membranes.

### Effects of MAE-HB unfilled resins on expression of *glucosyltransferase B (gtfB), glucosyltransferase C (gtfC) and glucosyltransferase D (gtfD)* in *S. mutans*

Glucosyltransferase is mainly responsible for the synthesis of water-insoluble glucans, which promote the adhesion of *S. mutans* to teeth and dental materials^24^. Thus, we evaluated glucosyltransferase gene expression by real-time quantitative PCR^25^,^26^. Briefly, specimens were placed in a 6-well plate to generate biofilms for RNA analysis. One hundred microliters of *S. mutans* suspension was added to each well containing 5 ml BHI supplemented with 1% sucrose. After incubating in an anaerobic atmosphere at 37 °C for 24 h, the unfilled resins were rinsed with phosphate buffered saline (PBS: 0.01 M, pH 7.2) to remove unattached cells, and *S. mutans* attached to the surface of specimens was collected for RNA extraction^27^. For cDNA synthesis, RNA was reverse transcribed using a QuantScript RT Kit (Tiangen Biotech Co., Beijing, China). Real-time quantitative PCR was employed to determine gene expression in a ABI7500 Real-Time PCR System (Applied Biosystems, Foster City, CA) using SYBR Green RealMasterMix (Tiangen Biotech CO., Beijing, China) according to the manufacturer’s instructions. The primers for real-time PCR are shown in Table 1. Gene expression was normalized to 16S rRNA. Five separate experiments were performed for each group.

### Statistical analysis

The antibacterial activities of immobilized MAE-HB and released MAE-HB at different immersion times were compared by Kruskal–Wallis H test and Mann–Whitney U test. Real-time RT-PCR results were analysed using a two-sample t-test. Statistical analyses were performed with SPSS 14.0 software and significance level was set at P < 0.05.

## Results

### Antibacterial activity of immobilized MAE-HB

**Figure 2 f2:**
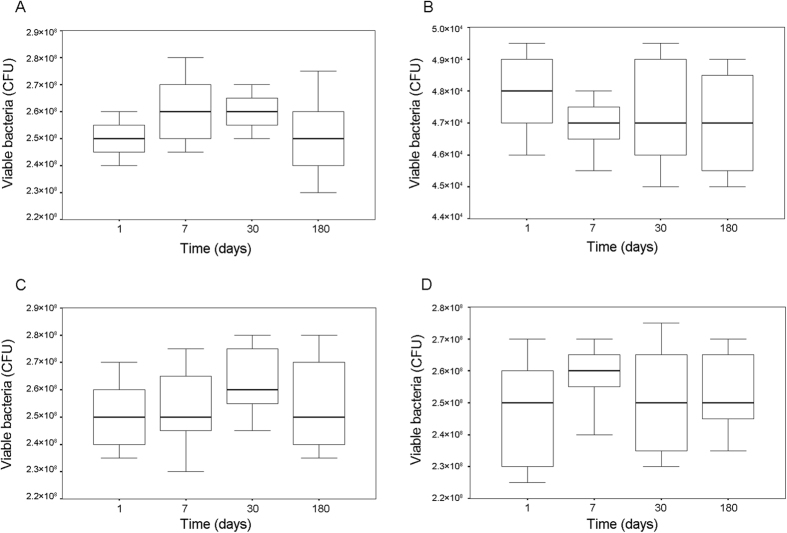
Antibacterial activities of immobilized MAE-HB and released MAE-HB after different immersion times. (**A**) The antibacterial activities of 0%-MAE-HB unfilled resins. (**B**) The antibacterial activities of 10%-MAE-HB unfilled resins. (**C**) The antibacterial activities of eluents from 0%-MAE-HB unfilled resins. (**D**) The antibacterial activities of eluents from 10%-MAE-HB unfilled resins.

The antibacterial activities of 0%- and 10%-MAE-HB unfilled resins at different immersion times are shown in Fig. 2A,B, respectively. Compared with the control group, bacterial growth was suppressed significantly on 10%-MAE-HB unfilled resins at 1 d, 7 d, 30 d, or 180 d (P < 0.05). The time of incubation with the 10%-MAE-HB unfilled resins had no significant difference on antibacterial activity (P > 0.05).

### Antibacterial activity of released MAE-HB

The antibacterial activities of eluents from 0%- and 10%-MAE-HB unfilled resins are shown in Fig. 2C[Fig f2]D, respectively. No significant differences were found between the two groups at any time point (P > 0.05).

### Fe-SEM observation

**Figure 3:**
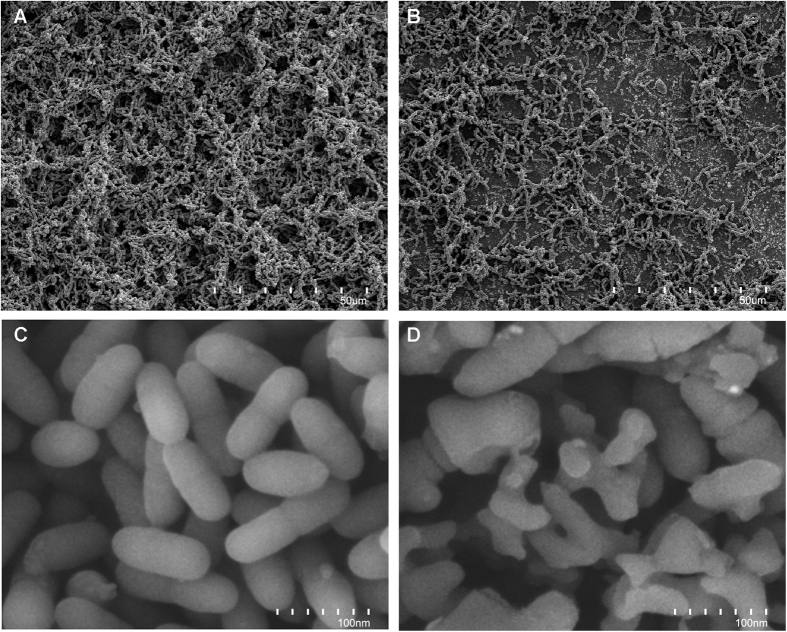
Fe-SEM images of *S. mutans* accumulation after anaerobic inoculation with (**A**) 0%- MAE-HB unfilled resin and (**B**) 10%-MAE-HB unfilled resin. Fe-SEM images of *S. mutans* morphology after anaerobic inoculation with (**C**) 0%- MAE-HB unfilled resin and (**D**) 10%-MAE-HB unfilled resin.

The Fe-SEM observations show that, after anaerobic growth at 37 °C for 4 h, a significant amount of *S. mutans* accumulated on the surface of 0%-MAE-HB unfilled resin (Fig. 3A). In contrast, a small amount of *S. mutans* was found on the surface of 10%-MAE-HB unfilled resin (Fig. 3B). High magnification images revealed a normal morphology of *S. mutans* on 0%-MAE-HB unfilled resin (Fig. 3C), but a disturbed integrity of bacterial cells on 10%-MAE-HB unfilled resin (Fig. 3D).

### CLSM analysis of bacterial growth

**Figure 4:**
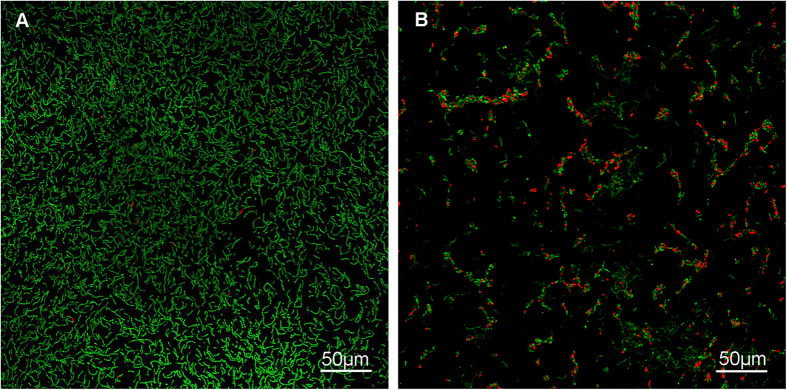
Representative confocal laser-scanning microscope (CLSM) images of live/dead-stained *S. mutans* after anaerobic inoculation with (**A**) 0%- MAE-HB unfilled resin and (**B**) 10%-MAE-HB unfilled resin. Live bacteria exhibited green fluorescence, and bacteria with compromised membranes exhibited red fluorescence. Scale bars, 50 μm.

Representative live/dead staining CLSM images of the adherent biofilms on resin disks are shown in Fig. 4. The specimen of the 0%-MAE-HB unfilled resin was fully covered by primarily live bacteria (green), whereas the 10%-MAE-HB unfilled resin showed a lower density of cells and a greater proportion of dead bacteria (red).

### Effects of MAE-HB unfilled resins on expression of *glucosyltransferase B (gtfB), glucosyltransferase C (gtfC) and glucosyltransferase D (gtfD)* in *S. mutans*

**Figure 5:**
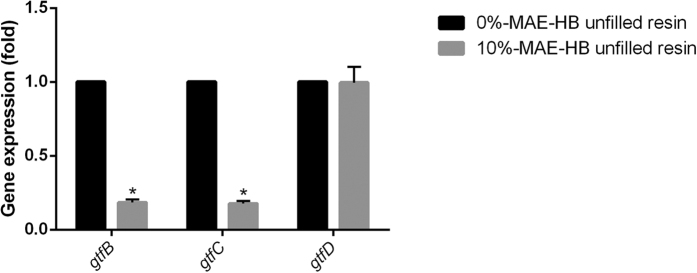
The expression of *gtfB, gtfC*, and *gtfD* by *S. mutans* on 0%- and 10%-MAE-HB unfilled resins. “*” denotes statistical significance (*P* < 0.05).

Figure 5 shows the expression of *gtfB, gtfC*, and *gtfD* in *S. mutans* biofilms attached to 0%- and 10%-MAE-HB unfilled resins. Both *gtfB* and *gtfC* expression on the 10%-MAE-HB unfilled resin was lower than that of the control group (P < 0.05), while no significant difference was found with respect to *gtfD* expression (P > 0.05).

## Discussion

Shrinkage of resins during the polymerization process is the main cause of secondary caries^6^. However, bacterial antigens and metabolic by-products can also diffuse through gaps between the resin and tooth, and may thereby lead to pulpal inflammation and infection^28^,^29^. Therefore, resins must be endowed with strong and long-lasting antibacterial properties in order to reduce the occurrence of secondary caries and protect pulp health.

Current antibacterial resin materials can be divided into agent-releasing and non-agent-releasing subtypes^22^. Soluble antimicrobials can easily be incorporated into resins to endow them with antibacterial activity. However, antibacterial agents are simply dispersed in resin matrix, and therefore their efficacy decreases with time. It is difficult to control the kinetics of release and, further exacerbating the problem, the release of soluble antimicrobials may exhibit unwanted side-effects against surrounding tissues and compromise the mechanical properties of composites^15^,^28^,^29^,^30^.

Non-agent-releasing antibacterial materials have been developed to overcome the disadvantages of agent-releasing antibacterial materials. A promising candidate is polymerizable QAS, which can polymerize with resin matrix, and exerts long-lasting antibacterial activity against a wide range of bacteria, fungi, and viruses^17^,^22^. Two typical examples of polymerizable QAS are MDPB, which was developed by Imazato *et al*.^17^, and DMAE-CB^31^, which was developed by our research group. These materials contain MDPB and DMAE-CB monomers that are chemically bound to the resin matrix, from where they exert stable antibacterial activity. However, there is only one double bond in the chemical structures of MDPB and DMAE-CB; thus only a small amount of MDPB and DMAE-CB can polymerize with the resin matrix, which limits their antibacterial activities^19^,^21^.

To increase the amount of polymerizable antibacterial monomer incorporated into resin materials, our research group developed the novel polymerizable QAS monomer, MAE-HB. The MAE-HB monomer contains two double bonds and can therefore polymerize with resin matrix more completely than previous substrates. MAE-HB exhibits strong bactericidal action against oral bacteria, and is rapidly bactericidal against *S. mutans*. At a concentration of 48.8 mg/ml (4 × MBC), MAE-HB kills 99.99% of *S. mutans* within 1 min of incubation, and no viable bacteria are detected after 30 min^22^. Thus, MAE-HB is a good candidate for conferring antibacterial properties upon resin materials.

*S. mutans* is one of the major pathogens responsible for human dental caries, and is therefore often used for investigations into the antibacterial effects of modified unfilled resins^19^. Our current study indicates that 10%-MAE-HB unfilled resins have strong antibacterial effects against *S. mutans*, even after immersion in water for 180 days. In order to verify that the strong and long-lasting antibacterial effect was due to immobilized MAE-HB rather than released material, the eluent from 10%-MAE-HB unfilled resins was compared with that of control at all time points. The eluent had no antibacterial activity, and virtually no MAE-HB monomer was released from unfilled resins, even after immersion in water for 180 days. Thus, we infer that the quaternary ammonium group endowed the MAE-HB monomer with strong antibacterial activity, and that the dimethacrylate groups helped immobilize MAE-HB monomers in the resin matrix through covalent bonding after curing^26^.

The Fe-SEM findings and CLSM findings were concordant, as they revealed that the death induced by the 10%-MAE-HB unfilled resin correlated with profound perturbations to bacterial morphology. Polymerizable QAS exhibits antibacterial activity due to its ability to adsorb negatively charged bacterial cells onto a positively charged quaternary amine N^+ ^32,33,34. This process may contribute to the disruption of cell membranes, disturb the electric balance, and subsequently lead increased cell permeability, which ultimately may cause bacterial cell lysis^32^,^33^,^34^,^35^,^36^.

*S. mutans* produces glucosyltransferases, and subsequently synthesizes glucans *in situ*, which provides binding sites for cariogenic microorganisms, leading to dental plaque formation^24^,^37^. In addition, glucosyltransferases adsorbed to the surfaces of other oral microorganisms may convert them to glucan producers^24^. At least 3 *gtf* genes are involved in this process; *gtfB* encodes GTFB enzyme that synthesizes primarily insoluble glucan, *gtfC* encodes GTFC enzyme that produce a mixture of soluble and insoluble glucans, and *gtfD* encodes GTFD enzyme that forms predominantly soluble glucans^37^. Among them, the activities of GTFB and GTFC are the most important for building the biofilm structure^38^,^39^ and water-insoluble glucans are the main composition of extracellular polysaccharides in dental biofilm, while GTFD serve as primers for GTFB and as a reserve source of energy and contribute in part at least to the low pH values observed in cariogenic plaque^37^,^40^,^41^,^42^. Decreased expression of *gtf* genes could reduce glucan synthesis, which would in turn reduce bacterial adhesion^25^.

In the present study, we found that *gtfBC* expression was significantly lower in the presence of 10%-MAE-HB unfilled resin, while *gtfD* expression was unaffected. Consistent with this, another QAS monomer, methacryloxylethyl cetyl dimethyl ammonium chloride (DMAE-CB), also has an inhibitory effect on the expression of these enzymes in *S. mutans* biofilms^25^. *gtf* gene expression can be influenced by a variety of reasons, such as carbohydrate availability and source, environmental pH, and growth phase/rate^43^,^44^,^45^,^46^,^47^. Thus we speculate that the suppression of *gtfBC* may be related to the chemical structure of QAS, and we suggest that the cationic moiety of MAE-HB could attract negatively charged exopolysaccharides of *S. mutans*. This may alter the physicochemical properties of the exopolysaccharide and the milieu for bacterial growth, which in turn could inhibit *gtfB* and *gtfC* expression^25^,^37^. Besides, previous studies have shown that the *gtfB* and *gtfC* genes are in an operon-like arrangement and may have a common promoter and appear to be coordinately expressed^43^. *gtfD* gene is located upstream of *gtfBC* loci, presents an independent promoter and may be regulated in a manner opposite that of *gtfB* and *gtfC*^48^,^49^. Thus we speculate that, *gtfB* and *gtfC* can be cotranscribed and be subjected to the same regulatory mechanisms when MAE-HB is present, as described in other studies^48^,^50^,^51^,^52^. Moreover, Ooshima *et al*.^53^ found that when the level of bacterial adherence was associated with the ratio of 3 different GTFs. Taken together, the reduced *gtfBC* expression and unaltered *gtfD* expression by10%-MAE-HB unfilled resin would alter the ratio of GTFs, hence disturbing glucan synthesis, preventing biofilm attachment and cariogenic bacteria accumulation.

In summary, this study indicates that the incorporation of MAE-HB endows unfilled resins with strong and long-lasting antibacterial effects against *S. mutans*, and could therefore play an important role in preventing the occurrence of secondary caries. This antibacterial effect was mainly caused by the positively charged quaternary amine N^+^ within MAE-HB. In addition, 10%-MAE-HB unfilled resin inhibits bacterial adhesion by modulating the expression of glucosyltransferases. This might facilitate the inhibition of caries via blockade of cariogenic biofilm accumulation. We suggest that MAE-HB is a promising candidate for incorporation in to dental resins with potent antibacterial activity.

## Additional Information

**How to cite this article**: Huang, L. *et al*. Antibacterial activity of a modified unfilled resin containing a novel polymerizable quaternary ammonium salt MAE-HB. *Sci. Rep.* **6**, 33858; doi: 10.1038/srep33858 (2016).

## Figures and Tables

**Table 1:** Primer sequences used for qRT-PCR analysis.

Gene	Accession number	Forward primer sequences	Reverse primer sequences
*gtfB*	M17361	AGCAATGCAGCCAATCTACAAAT	ACGAACTTTGCCGTTATTGTCA
*gtfC*	M22054	CTCAACCAACCGCCACTGTT	GGTTTAACGTCAAAATTAGCTGTATTAGC
*gtfD*	M29296	ACAGCAGACAGCAGCCAAGA	ACTGGGTTTGCTGCGTTTG
*16S*	X58303	CCTACGGGAGGCAGCAGTAG	CAACAGAGCTTTACGATCCGAAA
